# Machine learning-based clinical decision support system for treatment recommendation and overall survival prediction of hepatocellular carcinoma: a multi-center study

**DOI:** 10.1038/s41746-023-00976-8

**Published:** 2024-01-05

**Authors:** Kyung Hwa Lee, Gwang Hyeon Choi, Jihye Yun, Jonggi Choi, Myung Ji Goh, Dong Hyun Sinn, Young Joo Jin, Minseok Albert Kim, Su Jong Yu, Sangmi Jang, Soon Kyu Lee, Jeong Won Jang, Jae Seung Lee, Do Young Kim, Young Youn Cho, Hyung Joon Kim, Sehwa Kim, Ji Hoon Kim, Namkug Kim, Kang Mo Kim

**Affiliations:** 1 Department of Radiation Oncology, Korea University Guro Hospital, Korea University College of Medicine, Seoul, Republic of Korea; 2 Department of Internal Medicine, Seoul National University Bundang Hospital, Seoul National University, Seongnam, Republic of Korea; 3 Department of Radiology and Research Institute of Radiology, Asan Medical Center, University of Ulsan College of Medicine, Seoul, Republic of Korea; 4 Department of Gastroenterology, Asan Liver Center, Asan Medical Center, University of Ulsan College of Medicine, Seoul, Republic of Korea; 5 Department of Internal Medicine, Samsung Medical Center, Seoul, Republic of Korea; 6 Department of Internal Medicine, Inha University Hospital, Incheon, Republic of Korea; 7 Department of Internal Medicine, Seoul National University Hospital, Seoul National University, Seoul, Republic of Korea; 8 Department of Internal Medicine, Seoul St. Mary’s Hospital, Seoul, Republic of Korea; 9 Department of Internal Medicine, Incheon St. Mary’s Hospital, Incheon, Republic of Korea; 10 Department of Internal Medicine, Seoul Severance Hospital, Seoul, Republic of Korea; 11 Department of Internal Medicine, Chung-Ang University Hospital, Seoul, Republic of Korea; 12 Department of Internal Medicine, Korea University Guro Hospital, Korea University College of Medicine, Seoul, Republic of Korea; 13 Department of Internal Medicine, Bundang Jesaeng General Hospital, Seongnam, Republic of Korea; 14 Department of Convergence Medicine, Asan Medical Center, University of Ulsan College of Medicine, Seoul, Republic of Korea

**Keywords:** Disease-free survival, Liver cancer

## Abstract

The treatment decisions for patients with hepatocellular carcinoma are determined by a wide range of factors, and there is a significant difference between the recommendations of widely used staging systems and the actual initial treatment choices. Herein, we propose a machine learning-based clinical decision support system suitable for use in multi-center settings. We collected data from nine institutions in South Korea for training and validation datasets. The internal and external datasets included 935 and 1750 patients, respectively. We developed a model with 20 clinical variables consisting of two stages: the first stage which recommends initial treatment using an ensemble voting machine, and the second stage, which predicts post-treatment survival using a random survival forest algorithm. We derived the first and second treatment options from the results with the highest and the second-highest probabilities given by the ensemble model and predicted their post-treatment survival. When only the first treatment option was accepted, the mean accuracy of treatment recommendation in the internal and external datasets was 67.27% and 55.34%, respectively. The accuracy increased to 87.27% and 86.06%, respectively, when the second option was included as the correct answer. Harrell’s C index, integrated time-dependent AUC curve, and integrated Brier score of survival prediction in the internal and external datasets were 0.8381 and 0.7767, 91.89 and 86.48, 0.12, and 0.14, respectively. The proposed system can assist physicians by providing data-driven predictions for reference from other larger institutions or other physicians within the same institution when making treatment decisions.

## Introduction

In 2020, primary liver cancer was the sixth most commonly diagnosed cancer and the third leading cause of cancer-related deaths worldwide^1^. The majority of liver cancers are hepatocellular carcinomas (HCC). Given that most HCC patients also suffer from liver dysfunction, the benefits of treating the cancer must be weighed against the potential harms of medical interventions. Consequently, treatment decisions for HCC patients are highly multifactorial, with physicians taking into account not only the tumor burden but also the extent of liver dysfunction and performance status. The Barcelona Clinic Liver Cancer (BCLC) algorithm, a widely used staging system^2^, has been endorsed in clinical practice guidelines^3,4^. However, there is a significant difference in the initial treatment choice between the recommendations of the BCLC system and real-world clinical practice, particularly for patients in East Asia^5,6^. One of the possible reasons for this discrepancy is etiological and ethnic differences, which play an important role in post-treatment prognosis, as well as differences in preferred treatment and medical reimbursement plans.

A clinical decision support system (CDSS) is an information system designed to improve healthcare delivery by enhancing medical decisions with targeted clinical knowledge, patient information, and other health information^7–9^. Recently, artificial intelligence (AI) has been applied to CDSS to predict post-treatment prognosis for patients using machine learning (ML) or artificial neural networks^10,11^. Previously, we developed ML-based CDSS to recommend an initial treatment option and predict post-treatment survival for HCC patients^12^. This model performed well in an institutional patient cohort. In this study, we externally validated the previous model using multi-center datasets from eight institutions in South Korea and made modifications to ensure its effective utilization across multiple institutions.

## Results

### Demographic and baseline clinical characteristics of patients

Table 1 presents the demographic and baseline clinical characteristics for all datasets. A full list of patient characteristics for each center in the external validation datasets is described in the Appendix (pp 10–12). Excluding patients who underwent other therapies and transplantation, the final study populations for the internal and external validation datasets included 935 and 1750 patients, respectively. Initial treatment options included radiofrequency ablation or percutaneous ethanol injection therapy (RFA or PEIT) in 6.8% to 21.6% of patients, resection in 3.7% to 35.8%, trans-arterial chemoembolization (TACE) in 34.8% to 64.8%, TACE combined with external beam radiotherapy (EBRT) in 0% to 24.4%, sorafenib treatment in 0% to 7.4%, and supportive care in 3.1% to 16.7% of patients.

**Table 1:** Baseline characteristics of the patients in the internal and external validation datasets.

		Internal dataset (*n* = 935)	External validation dataset (*n* = 1750)	*p* value
Age, year		56.9 (24–91)	59.2 (27–93)	<0.0001
Gender	Male	764 (81.7)	1412 (80.7)	0.5524
	Female	171 (18.3)	338 (19.3)	
ECOG performance status	0	416 (44.5)	984 (56.2)	<0.0001
	1 or 2	481 (51.4)	716 (40.9)	
	3 or 4	38 (4.1)	50 (2.9)	
Ascites	Absent	787 (84.2)	1403 (80.2)	0.0126
	Present	148 (15.8)	347 (19.8)	
Varices	Absent	667 (71.3)	1048 (59.9)	<0.0001
	Present	268 (28.7)	702 (40.1)	
Child-Pugh class	A	728 (77.9)	1353 (77.3)	0.7533
	B	178 (19.0)	333 (19.0)	
	C	29 (3.1)	64 (3.7)	
Body mass index, kg/m^2^		24.1 (11.7–41.2)	24.1 (13.8–41.2)	0.4269
Tumour number	1	561 (60.0)	1040 (59.4)	0.4916
	2 or 3	180 (19.3)	368 (21.0)	
	≥4	194 (20.7)	342 (19.5)	
Maximal tumour size, cm		5.2 (0.5–10.0)	4.7 (0.7–10.0)	<0.0001
Distribution	Single segmental	447 (47.8)	870 (49.7)	0.2699
	Unilobar	221 (23.6)	431 (24.6)	
	Bilobar	267 (28.6)	449 (25.7)	
Distant metastasis	Absent	823 (88.0)	1594 (91.1)	0.014
	Present	112 (12.0)	156 (8.9)	
Vascular invasion	Absent	713 (76.3)	1323 (75.6)	0.0002
	Unilateral	146 (15.6)	209 (11.9)	
	Main or bilateral	76 (8.1)	218 (12.5)	
RFA feasibility^b^	Feasible	193 (20.6)	479 (27.4)	0.0002
	Non-feasible	742 (79.4)	1271 (72.6)	
Laboratory findings	AFP^a^, ng/mL	42.1 (0.4 – 2,689,770)	33.1 (0.7 – 1,339,065)	0.0734
	Hemoglobin, g/dL	13.4 (6.2–19.6)	13.3 (3.3–22.0)	0.4460
	Platelet count, x10^9^/mm^3^	159.9 (13.5–706.0)	148.2 (0.8–640.0)	<0.0001
	ALT, U/L	51.1 (4–1914)	51.7 (0.1–1135)	0.2153
	Total bilirubin, ml/dL	1.5 (0.3–42.9)	1.4 (0.2–38.2)	0.0005
	Albumin, mg/dL	3.6 (1.1–5.0)	3.8 (0.8–5.2)	<0.0001
	Prothrombin time, INR	1.1 (0.8–2.8)	1.1 (0.8–12.9)	<0.0001
	Creatinine, mg/dL	0.9 (0.2–11.4)	0.9 (0–12.3)	<0.0001
Initial treatment	RFA or PEIT	78 (8.3)	240 (13.7)	<0.0001
	Resection	335 (35.8)	289 (16.5)	
	TACE	325 (34.8)	889 (50.8)	
	TACE combined with EBRT	65 (7.0)	114 (6.5)	
	Sorafenib treatment	30 (3.2)	60 (3.4)	
	Supportive care	102 (10.9)	158 (9.0)	

Data are *n* (%), mean or ^a^median (range) in parentheses. *p* values were calculated using the χ^2^ test or Student t-test or Mann–Whitney U test.

*AFP* alpha-fetoprotein. *ALT* alanine aminotransferase. *EBRT* external beam radiotherapy. *ECOG* Eastern Cooperative Oncology Group. *INR* international normalized ratio. *PEIT* percutaneous ethanol injection. *RFA* radiofrequency ablation. *TACE* transarterial chemoembolization.

^b^ RFA feasibility was defined as the size or location of the tumor to receive percutaneous RFA successfully without significant complications.

### Treatment recommendation model for multi-center setting

The seven top-performing classifiers, sorted by accuracy were the C-support vector machine (SVM) with linear kernel, exhibiting the highest mean accuracy and recall, followed by the Gaussian process, random forest, extra-trees, histogram-based gradient boosting, light gradient boosting machine, and multi-layer perceptron classifiers (Table 2). However, the SVM alone performed worse than the ensemble voting machines that included three to seven top-performing classifiers. There were no significant differences between voting classifiers according to the number of top-performing classifiers (Appendix p 15). Compared to the previous cascaded model^12^, the mean accuracy of the ensemble voting machine increased for both internal and external datasets (Appendix p 15). The mean accuracy of the external validation using ensemble voting machine was 55.34%, which was lower than that of the internal dataset at 67.27%.

**Table 2:** Top 7 classifiers sorted by accuracy for internal dataset.

Model	Accuracy	Recall	Precision	F_1_ score	Kappa score	MCC
Linear SVM	65.45 (3.49)	51.09 (4.26)	64.79 (3.71)	64.32 (3.20)	51.41 (4.79)	51.75 (4.93)
GPC	64.71 (2.66)	48.92 (3.24)	63.05 (2.50)	63.22 (2.49)	50.02 (3.73)	50.41 (3.80)
RF	64.49 (5.17)	47.54 (5.18)	62.97 (6.04)	63.01 (5.19)	49.48 (7.27)	49.92 (7.53)
ET	64.49 (3.18)	49.83 (5.00)	63.29 (3.41)	63.37 (3.21)	50.07 (4.77)	50.37 (4.85)
HGBC	64.28 (2.99)	49.07 (5.62)	64.02 (3.58)	63.22 (2.80)	49.79 (4.32)	50.20 (4.54)
Light GBM	63.74 (3.05)	49.62 (4.26)	62.81 (2.71)	62.73 (2.65)	49.38 (4.17)	49.73 (4.41)
MLP	63.64 (3.04)	48.72 (2.88)	62.44 (2.10)	62.46 (2.58)	48.63 (4.31)	48.88 (4.35)

Data are mean (SD) in parentheses.

*MCC* Matthew’s correlation coefficient. *Linear SVM* C-support vector machine with linear kernel. *GPC* gaussian process classifier. *RF* random forest classifier. *ET* extra-trees classifier. *HGBC* histogram-based gradient boosting classifier. *Light GBM* light gradient boosting machine. *MLP* multi-layer perceptron classifier.

The results of individual training with each institutional dataset demonstrated higher mean accuracy and recall compared to those of external validation in all centers except for one, Severance Hospital (Appendix p 16). The mean accuracy for each institutional dataset was 66.53%, which was higher than that of the external validation, 55.34%. Table 3 shows the performance of the ensemble voting machine when the second treatment option is considered correct in both internal and external datasets. When the second option was considered correct, the accuracy of external validation increased to 86.06%, similar to that of the internal dataset and individual training for the external datasets. After model calibration, there was a slight decrease in mean accuracy, which was more pronounced in the external validation datasets (Appendix pp 18–19). The decrease in accuracy can be attributed to significant internal variations and diversity within the datasets. Conversely, the standard deviation decreased after model calibration, indicating that calibration contributed to more consistent results.

**Table 3:** Comparison of performance considering second treatment options.

Treatment options	Internal dataset		External datasets			
			External validation		Individual training	
	Accuracy	Recall	Accuracy	Recall	Accuracy	Recall
1^st^ option	67.27 (2.94)	52.22 (4.67)	55.34 (6.09)	41.68 (3.88)	66.53 (5.90)	46.56 (8.13)
2^nd^ option	87.27 (2.25)	71.24 (2.90)	86.06 (3.10)	64.49 (8.16)	84.08 (2.55)	59.63 (6.83)

Data are mean (SD) in parentheses.

### Prediction of individual post-treatment survival and risk stratification for each treatment

**Fig. 1:**
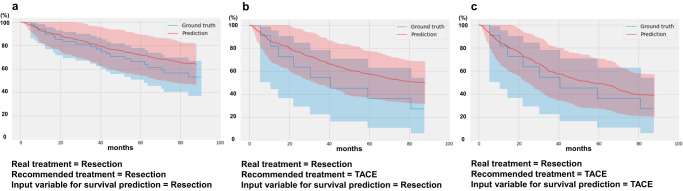
Actual (=Ground truth) and predicted (=Prediction) survival curves according to a treatment input variable in the second-stage model. **a** Patients who were recommended resection in the first-stage model and actually underwent the resection. In the second-stage model, the treatment input variable was set as resection. **b**, **c** Patients who were recommended TACE in the first-stage model but underwent resection in actual practice. In the second-stage model, the treatment input variable was set as resection in **b** and TACE in **c**.

**Fig. 2:**
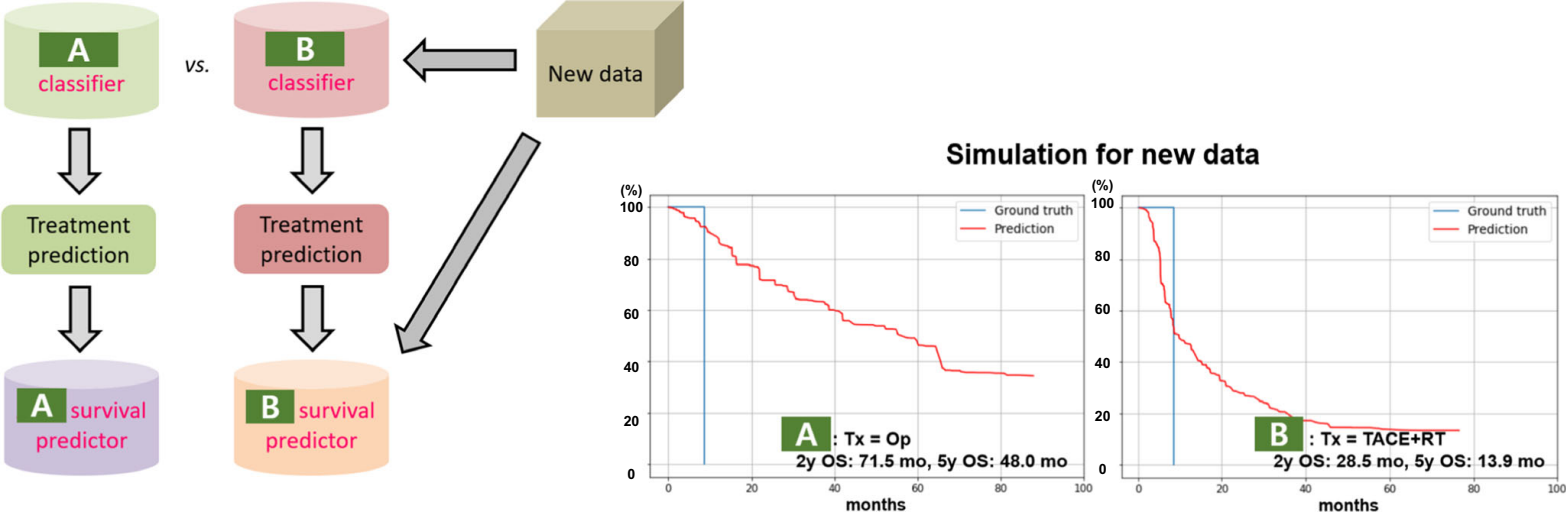
An example of simulations on the new data using models trained individually with each institutional dataset.

Table 4 displays the performance of the survival prediction model, which achieved better results in the internal dataset compared to the external validation dataset. The integrated time-dependent area under the receiver operating characteristic curve (iAUC) ranges from 72.63 to 86.18, with TACE showing the best performance. Figure 1 provides simulation examples of risk stratification for specific treatments using the model. In the group of patients who actually underwent resection, the actual survival rate of those recommended for resection by the first-stage model (Fig. 1a) was higher compared to the group recommended for TACE (Fig. 1b, c). Moreover, among patients who were initially recommended for TACE by the first-stage model but actually underwent resection (Fig. 1b, c), the survival prediction curves based on setting TACE as the treatment input variable in the survival prediction model showed a stronger correlation with the actual survival curves compared to those for resection. These findings demonstrate that our model has the capability to predict the prognosis of patients accurately. In the supplementary experiments, we used propensity score matching to achieve a closer match between the two groups. We then evaluated the model’s performance and analyzed the reasons for the observed changes in its performance (Appendix pp 20–25). Furthermore, we used models trained separately with two institutional datasets to simulate different treatment choices and subsequent survival prediction outcomes for new data from another center as shown in Fig. 2.

**Table 4:** Performance of survival prediction of internal and external datasets.

Dataset	Center	Performance		
		C-index	iAUC	IBS
Internal dataset	AMC	0.8381 (0.0276)	91.89 (2.08)	0.12 (0.01)
External datasets	KUGH	0.7812	87.08	0.10
	SNUBH	0.7833	88.23	0.15
	SMC	0.7580	84.28	0.15
	SNUH	0.8053	89.48	0.14
	CMC	0.7458	84.78	0.18
	SH	0.8254	89.39	0.10
	CUH	0.7208	80.73	0.17
	IUH	0.7935	87.84	0.10
	Average	0.7767 (0.0315)	86.48 (2.82)	0.14 (0.03)

Data are mean (SD) in parentheses.

*C-index* Harrell’s concordance index. *iAUC* integrated time-dependent area under the receiver operating characteristic curve. *IBS* integrated Brier score. *KUGH* Korea University Guro Hospital. *SNUBH* Seoul National University Bundang Hospital. *SMC* Samsung Medical Center. *SNUH* Seoul National University Hospital. *CMC* Catholic Medical Center. *SH* Severance Hospital. *CUH* Chung-ang University Hospital. *IUH* Inha University Hospital.

## Discussion

In this study, we developed a two-stage ML-based model for treatment recommendation and post-treatment survival prediction for patients with HCC, which was validated using multi-center datasets. The performance of external validation on treatment recommendation using the ensemble voting machine was inferior to the results obtained from the internal dataset and those trained individually with each institutional dataset. The cause of this performance decline is speculated to be due to the different treatment options among institutions, prompting us to propose a second treatment option in addition to the first, using the probabilities from the ensemble voting machine. Furthermore, the risks associated with each treatment can be stratified by predicting individual survival based on the recommended treatment and other prognostic variables.

The strength of our CDSS model lies in its ability to not only provide treatment suggestions but also prognoses associated with those treatments. This model can serve as a supplementary system to the current guidelines, assisting physicians in determining treatment options for HCC. Additionally, it can help explain the rationale behind treatment choices to patients. In particular, treatment selection can be further supported by predicting individualized survival graphs for patients with various treatment options available or who are at the borderline of the guidelines. Furthermore, this model can be beneficial in aiding treatment decisions for inexperienced doctors or in situations where multidisciplinary care poses challenges.

However, developing a CDSS for treatment decisions in HCC presents significant challenges. One of the primary factors contributing to these challenges is the intricacy of determining the optimal treatment option due to the heterogeneous nature of HCC and underlying chronic liver disease. The optimal treatment option for HCC may vary depending on individual patients’ medical conditions, insurance coverage, and willingness to undergo treatment. Consequently, the management of HCC in real clinical practice often deviates from the treatment options recommended by the widely accepted BCLC staging system^13,14^. In this study, we developed a CDSS model that emulates treatment decisions made by clinical physicians to address the substantial disparities observed between actual treatment decisions and the recommended treatment guidelines. However, one of the most significant issues associated with this type of CDSS is the rationality and appropriateness of the judgments made by the clinical physicians used as the reference standard. To mitigate these concerns, we provided alternative options and corresponding survival rates in addition to the optimal treatment option. Moreover, we recommend the complementary use of a data-driven CDSS system based on the recommendation-based current guidelines.

Another notable limitation of the current data-driven CDSS is its inherently data-restrictive nature. Over the past several decades, cancer treatment has undergone significant advancements, and the expansion of extensive genetic and clinical databases, supported by efficient computing systems, has accelerated the pace of treatment advances and shortened the cycle for updating treatment guidelines. However, data-driven models ultimately rely on past databases to derive conclusions. Consequently, if newly developed treatment options are inadequately represented or absent in the training dataset, the CDSS model may fail to select those treatments or accurately predict patient survival. During the study period, Sorafenib, a protein kinase inhibitor with activity against many protein kinases including vascular endothelial growth factor receptors, platelet-derived growth factor receptors, and RAF kinases^15,16^, was the first-line recommended treatment for advanced-stage HCC according to the BCLC staging system. However, due to issues related to insurance coverage, Sorafenib was not widely utilized in actual clinical practice. Consequently, it was rarely recommended by the current model, and the reliability of treatment classification and survival prediction for that treatment was low. Similarly, newly incorporated treatments for advanced-stage HCC in the recent BCLC guidelines, such as Atezolizumab-Bevacizumab, Durvalumab-Tremelimumab, and Lenvatinib, were entirely absent from the training dataset. Therefore, the existing model is unable to predict these newly incorporated treatments.

To overcome these limitations, physicians must acknowledge the constraints of the system and consistently update it with new data, with the assistance of the model developer. The field of cancer treatment is a constantly evolving domain where new therapies emerge to replace previous treatment modalities, and innovative treatment approaches continue to arise. Therefore, it is crucial to periodically update the model with new data and analyze whether the results align with physicians’ opinions and the latest treatment guidelines (Appendix p 26). This periodic analysis should also include the variable selection process. In a previous study, we selected 20 key features from the initial 61 pretreatment variables based on the importance scores calculated from the automated classifier model^12^. Feature reduction helps to select the most influential features by providing unique knowledge for inter-class discrimination^17,18^. However, the excluded features in this process could be significant factors in determining treatment choices for certain patients within the internal dataset or patients in external datasets. Furthermore, the factors that determine treatment and subsequent prognosis are constantly changing as new diagnostic methods, genetic biomarkers, and novel treatments are developed. Therefore, it is essential to select new deterministic features, evaluate their prognostic effects, and retrain the model with the modified features for continuous learning. Automatic data updates from an electronic medical record database and tumor segmentation using deep learning techniques from computed tomography (CT) or magnetic resonance imaging (MRI) may be useful for this process^19,20^.

Recently, several studies have explored the use of ML or deep learning to predict the prognosis of HCC patients, incorporating radiomics, proteomics, and genomics^21–26^. However, it remains challenging to predict initial treatment choices or post-treatment survival based solely on radiomic or genomic features extracted from diagnostic images or tissues at a specific time point. Furthermore, the effective integration of high-dimensional data such as images or genomic profiles with more concise clinical variables remains an unresolved issue. In this study, we employed clinically established prognostic factors to predict initial treatment choices and post-treatment survival of HCC patients. However, further research is needed to explore whether the combination of radiomics, proteomics, and genomic features with these established prognostic factors can enhance the accuracy of predictions.

In machine learning, overfitting is a prevalent issue that occurs when a model becomes excessively specialized to the training data, resulting in degraded performance on new, unseen data. One of the reasons for the decreased performance of our model on external datasets is primarily due to its training on the internal dataset, which led to overfitting. Specifically, the diversity and variability in treatment choices among institutions for patients with similar characteristics inevitably contributed to this performance decline. There have been numerous approaches proposed to address the issue of overfitting^27,28^. Increasing the diversity of the training data by collecting various examples or using data augmentation techniques can enhance the generalization of the model. Moreover, reducing the model’s complexity by limiting the number of parameters and employing regularization techniques can effectively prevent overfitting. In this study, we improved the model performance by utilizing an ensemble voting approach instead of using a single machine learning algorithm. To reduce the complexity of the model, we minimized hyperparameter tuning for each algorithm. Furthermore, considering the significant variation in treatment patterns among different institutions, we proposed the inclusion of alternative treatment options in addition to providing only one treatment option that may be overfitted to the internal dataset. However, to significantly improve the performance of external validation datasets, it may be more effective to incorporate external datasets into the training dataset or conduct individual training.

Medicine is not only a science, but also a social and psychological subject. Wilkinson et al. argued that the majority of health states and events are so complex that we can only understand them probabilistically, and chance can never be predicted at the individual level^29^. Recent studies on Watson for Oncology, an AI assistant decision system developed by IBM Corporation with the help of top oncologists from Memorial Sloan Kettering Cancer Center, also commonly mention the system’s limitations and complementary roles^30,31^. Nonetheless, in an era of accelerated developments in new diagnostic methods, biomarkers, and treatments, selecting the appropriate treatment for individuals based solely on simple guidelines is becoming increasingly challenging. Therefore, by incorporating our system as a complementary resource for physician decision-making, along with the existing BCLC guidelines, can assist in guiding physicians to make more individualized treatment choices. Furthermore, our proposed system can be utilized as a virtual simulation tool for comparing institutional treatment choices and post-treatment prognoses in advance. Although digital twin technology is still in its early stages of development in the healthcare and medicine fields^32–34^, our system can serve as an example of its potential application.

In conclusion, we developed and validated an ML-based model that offers initial treatment recommendations and predicts post-treatment survival for HCC patients, utilizing multi-center datasets. Several experiments were conducted to ensure the model’s applicability in a multi-center setting, and we have addressed various issues and strategies for implementing this system within an actual clinical environment. Our proposed CDSS can assist the process of making treatment decisions for HCC patients by providing data-driven predictions in conjunction with existing guidelines.

## Methods

### Internal dataset and pre-treatment variables

For the purposes of training and internal validation, we used a prior dataset, which comprised 1021 newly diagnosed HCC patients at the Asan Medical Center in South Korea between January and October 2010^12^. All enrolled patients were diagnosed with HCC using liver protocol CT or MRI or liver biopsy in accordance with the current guidelines of the American Association for the Study of Liver Diseases^35^. Exclusion criteria were as follows: (a) patients with a history of prior HCC treatment (*n* = 356), (b) patients with metastatic liver cancer or secondary malignancies that might affect survival (*n* = 36), (c) patients with combined hepatocellular cholangiocarcinoma (*n* = 21), (d) patients with incidentally detected HCC after transplantation (*n* = 7), and (e) patients who underwent cytotoxic chemotherapy, other targeted agents (brivanib, sunitinib, erlotinib), or combined treatments (*n* = 83).

We retrospectively collected a total of 61 pre-treatment demographic, clinical, and imaging variables, initial treatment information, and survival status of 1021 HCC patients from an institutional database (Appendix p 4). To select the key variables, we used importance scores calculated by the previously described automated classifier models (Appendix pp 5–9), resulting in a selection of 20 variables, including 14 patient-related factors (age, body mass index, Child-Pugh class, presence of varix, presence of ascites, Eastern Cooperative Oncology Group score, hemoglobin level, platelet count, albumin level, prothrombin time, alanine aminotransferase level, total bilirubin level, creatinine level, and alpha-feto protein level) and 6 tumor-related factors (tumor number, maximal tumor size, tumor distribution, presence of portal vein invasion, presence of metastasis, and RFA feasibility)^12^. Overall survival was defined as the time between HCC diagnosis and death from any cause.

Previously, the initial treatment options were grouped into eight categories: RFA/PEIT, surgical resection, TACE, TACE combined with EBRT, sorafenib treatment, supportive care, transplantation, and other therapies that did not fit into the other seven predefined categories. In this study, we excluded other therapies and transplantation options due to significant heterogeneity among institutions in the other therapies group and large differences in the number of patients between institutions due to the severe shortage of deceased liver donors in the transplantation group, respectively. Furthermore, due to one institution among the eight external centers reporting a maximal tumor size of 10 cm for tumors larger than 10 cm, the maximal tumor size values for the remaining centers, including the internal dataset, were uniformly adjusted using the same rule, wherein any size exceeding 10 cm was recorded as 10 cm.

### External datasets

We collected datasets from eight institutions for external validation of the algorithm, including Korea University Guro Hospital (*n* = 138), Seoul National University Bundang Hospital (*n* = 193), Samsung Medical Center (*n* = 439), Seoul National University Hospital (*n* = 224), Catholic Medical Center (*n* = 162), Severance Hospital (*n* = 148), Chung-ang University Hospital (*n* = 171), and Inha University Hospital (*n* = 275). All datasets were compiled from a retrospective database of HCC patients diagnosed in each institution between January 2010 and December 2012. De-identified information on 20 pre-treatment key variables, initial treatment information, and survival status were obtained at the patient level. The protocols of this study were approved by the Institutional Review Board of Asan Medical Center (IRB number: 2017-0188) and the study was approved by each institutional review board of all the participating institutions. The requirement for informed consent from patients was waived due to the retrospective nature of the study. All methods were performed in accordance with the relevant guidelines and regulations.

### Model development

**Fig. 3:**
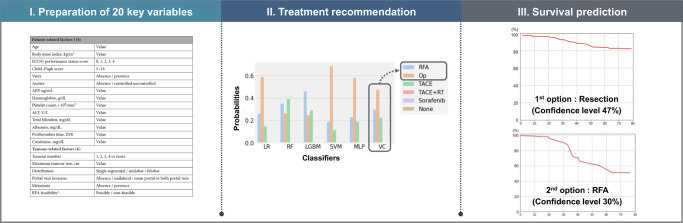
Overall workflow.

The ML algorithm consists of two stages: The first stage recommends initial treatment, and the second stage predicts post-treatment survival, as illustrated in Fig. 3. In the first stage, we modified the previous cascaded random forest model^12^ to an ensemble voting machine to improve the model’s flexibility and classification performance. Initially, we evaluated 19 ML algorithms for the development of the ensemble voting approach. Following the sorting of each classifier based on mean accuracy, the ensemble voting machine was trained using the top-performing three, five, and seven classifiers for the internal dataset. These ensemble voting machines were then compared with the top-performing classifier itself. Ultimately, we applied an ensemble voting machine involving the top five performing classifiers and evaluated its performance on both the internal and external validation datasets. Detailed information regarding the development of the ensemble voting machine is described in the Appendix (pp 13–15). Finally, we compared its performance to that of the previous cascaded random forest model.

We conducted two additional experiments to enhance the usability of this model in a multi-center setting. First, we trained the model using each institutional dataset (Individual training) and compared its performance with that of external validation. Second, we derived the first and second treatment options from the ensemble voting machine’s results using the highest and second-highest probabilities, respectively, and recalibrated the evaluation metrics by treating the second treatment option as the correct answer. The final performance of the ensemble model after model calibration was assessed in both the internal and external validation datasets. The process of model calibration is described in the Appendix (p 18). All experiments for the internal dataset and each institutional dataset were trained and tested using a five-fold cross-validation stratified by the treatment, while external validation was performed using the model trained with the entire internal dataset.

For the second stage, we trained the random survival forest algorithm with 21 variables, including 20 key variables and the initial treatment information, to predict post-treatment survival. Finally, we employed our two-stage model to simulate risk stratification for each treatment by predicting individual post-treatment survival based on the treatment recommendation results from the first stage model. TACE and resection, the two treatments with the highest proportion in the internal dataset, were used as illustrative examples for risk stratification.

### Statistical analysis

The baseline characteristics of patients between the internal dataset and the external datasets were compared using the chi-square test for categorical variables. For the continuous variables, we assessed normality using Shapiro-Wilk’s W test, and based on the results, we employed either the Student t-test or the Mann-Whitney U test for comparison. Per-patient based analysis was used to evaluate the treatment recommendation model, which included metrics such as accuracy, macro-average of recall, weighted-average precision, weighted-average *F*_1_ score, Cohen’s kappa score, and Matthew’s correlation coefficient^36^. For the survival prediction model, we utilized three metrics to evaluate performance: Harrell’s concordance index, iAUC, and integrated Brier score^37^. To evaluate the survival curve, we estimated it using a Kaplan-Meier fitter and compared it to those predicted by our model^38^. The open-source scikit-learn package version 0.23.2^39^, scikit-survival package version 0.15.0^40^, and scipy package version 1.5.4 were used for model development and statistical analyses.

### Supplementary information

Supplementary Information

## Data Availability

Limited deidentified data used for the analyses (internal and multi-center external datasets) in this work are available to qualified researchers upon request. Please email the corresponding author, Namkug Kim, Ph.D., at namkugkim@gmail.com.

## Code availability

The algorithms used in this study are publicly available at Github (https://github.com/mi2rl/hcc_cdss).
