# Supplementary material for: Machine learning-based clinical decision support system for treatment recommendation and overall survival prediction of hepatocellular carcinoma: a multi-center study
Source: NPJ Digit Med. 2024 Jan 5;7:2. doi: 10.1038/s41746-023-00976-8 (PMC10770025; doi:10.1038/s41746-023-00976-8)

SUPPLEMENTAL DATA

Machine learning-based clinical decision support system for treatment recommendation and overall survival prediction of hepatocellular carcinoma: a multi-center study

Table of contents

Feature selection **3**

Supplementary Table 1. Initially assembled 61 pretreatment variables. 4

Supplementary Figure 1. Feature importance (Top 23) from cascaded random forest models.. . 5

Supplementary Figure 2. Feature importance (Top 44) from random survival forest models 8

External validation datasets **10**

Supplementary Table 2. Baseline characteristics of the patients in the external validation datasets 10

Supplementary Figure 3. Treatment distribution patterns by participating institutions. 12

Model for treatment recommendation **13**

Supplementary Table 3. Performance in terms of the number of classifiers 15

Supplementary Table 4. Performance of ensemble voting classifier vs. cascaded random forest model. 15

Supplementary Table 5. Performance of individual training vs. external validation. 16

Model for survival prediction **17**

Calibration of model **18**

Supplementary Table 6. Performance of non-calibrated model vs. calibrated model 18

Supplementary Figure 4. Calibration plot. A) Pre-calibration. B) Post-calibration. 19

Propensity score matching **20**

Supplementary Table 7. Baseline characteristics of the patients 22

Supplementary Figure 5. Confusion matrix for the treatment classification model 24

Supplementary Figure 6. Predicted survival curve for the survival prediction model 25

Model deployment **26**

Supplementary Figure 7. Issues on model training and deployment 26

Feature selection

To enhance the performance of the model and improve its practical applicability in clinical settings, we conducted a feature reduction process, reducing the initially collected 61 pretreatment variables (Supplementary Table 1) to a final selection of 20 variables. In our previous study, we trained cascaded random forest classifiers to recommend each treatment and random survival forest models to predict survival rates after each treatment. During this process, the input variables were sorted based on the feature importance obtained from each model (Supplementary Figure 1-2).

Subsequently, two hepatologists (K.M.K with 22 years of experience and G.H.C with 9 years of experience in treating HCC patients), meticulously reviewed the sorted features and removed redundant variables, resulting in a final selection of 20 variables. Despite being ranked higher in terms of importance, certain variables were excluded for specific reasons. For example, "Presence of HV/IVC" and "bile duct invasion" were omitted due to the limited number of patients, and the "Millan criteria" were excluded as they could potentially overlap with the maximal tumor diameter and tumor number variables, making them redundant in the feature selection process.

Supplementary Table 1. Initially assembled 61 pretreatment variables.

**Patient variables (N = 30)**	**Laboratory variables (N = 13)**	**Tumor variables (N = 18)**
**Epidemiology**	WBC count	Enhancement pattern
Sex	Hemoglobin	Tumor type
Age	Platelet count	Tumor number
Performance status (ECOG)	PT (INR)	Maximal tumor diameter
Body mass index	Creatinine	Tumor distribution
	Estimated glomerular filtration rate	RFA feasibility[1)]
**Etiology**		Presence of dysplastic nodule
Alcohol history	Albumin	Presence of portal vein invasion
Amount of alcohol intake	AST	
Smoking history	ALT	Location of portal vein invasion
HBsAg	Total bilirubin	
HBeAg	AFP	Presence of hepatic vein invasion
HBeAb	PIVKA-II	
HBV DNA	ICG test	Presence of IVC invasion
History of HBV Treatment		Presence of bile duct invasion
HCV Ab		Presence of metastasis
HCV RNA		Presence of clinically significant metastasis
History of HCV treatment		
		Location of metastasis
**Liver cirrhosis-related**		BCLC stage
Child-Pugh class		Milan criteria
Varix		Asan criteria[2)]
Ascites		
Hepatic encephalopathy		
Presence of splenomegaly		
**Accompanying comorbidities**		
Hypertension		
Diabetes mellitus		
Dialysis		
Heart disease		
Pulmonary disease		
**Socio-economic status**		
Marriage		
Potential donor		
Occupation		
Education		
Residence area		

AFP, alpha-fetoprotein; *ALT*, alanine transaminase; *AST*, aspartate transaminase; *BCLC*, Barcelona clinic liver cancer; *ECOG*, Eastern Cooperative Oncology Group; *HBsAg*, hepatitis B surface antigen; *HBeAg*, hepatitis B envelope antigen; *HBeAb*, hepatitis B envelope antibody; *HBV*, hepatitis B virus; *HCV*, hepatitis C virus; *ICG*, indocyanine green; *INR*, international normalized ratio; *IVC*: inferior vena cava; *PIVKA-II*, protein induced by vitamin K absence or antagonist II; *PT*, prothrombin time; *RFA*, radiofrequency ablation; *WBC*, white blood cell.

[1)] RFA feasibility was defined as a size or location of the tumor to receive percutaneous RFA successfully without significant complication.

[2)] Institutional criteria of Asan Medical Center for liver transplantation.

Supplementary Figure 1. Feature importance (Top 23) from cascaded random forest models. A) RFA/PEIT or Resection vs. Not RFA/PEIT or Resection. B) RFA/PEIT vs. Resection. C) TACE vs. Not TACE.

A) RFA/PEIT or Resection vs. Not RFA/PEIT or Resection

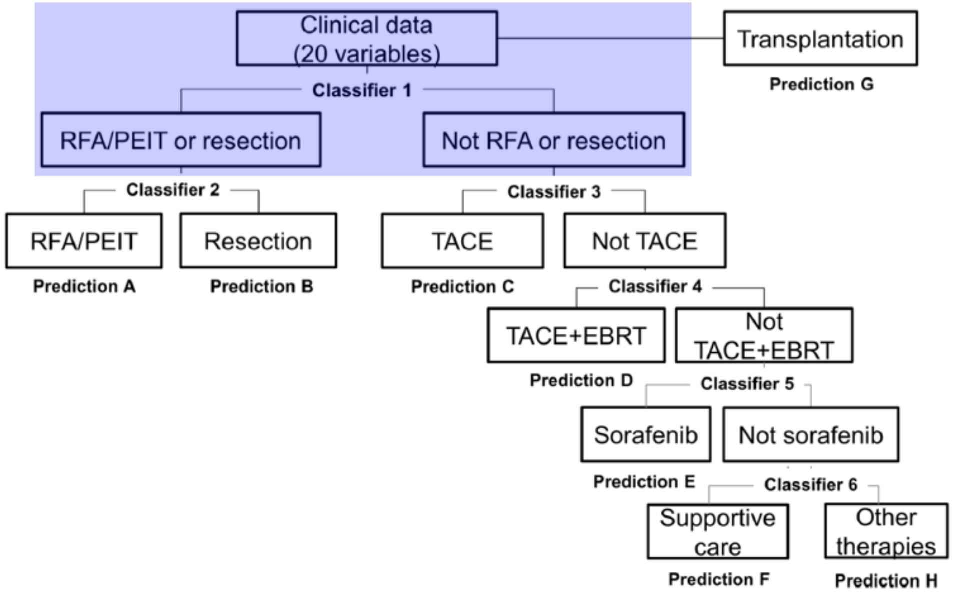

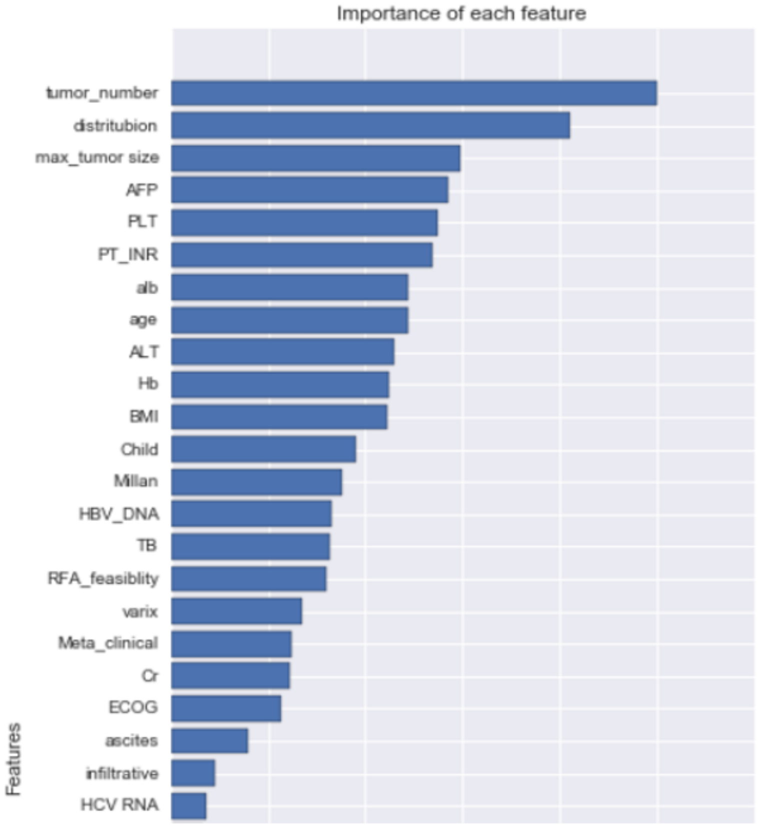

B) RFA/PEIT vs. Resection

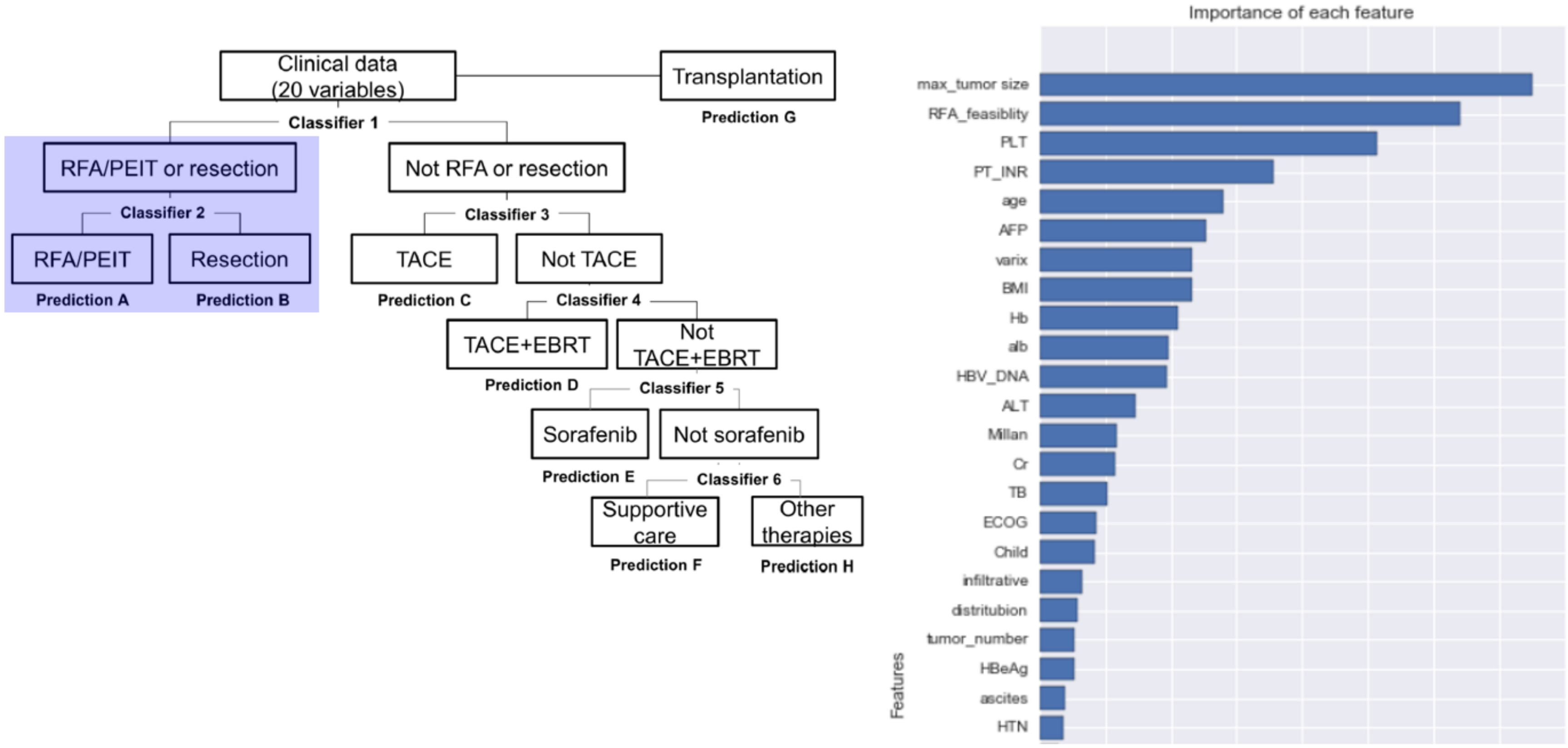

C) TACE vs. Not TACE

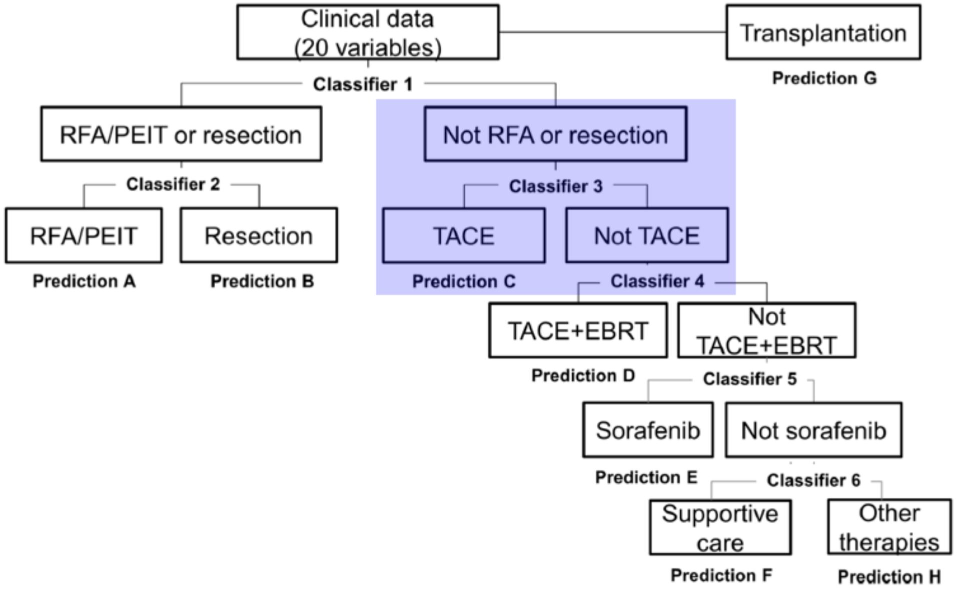

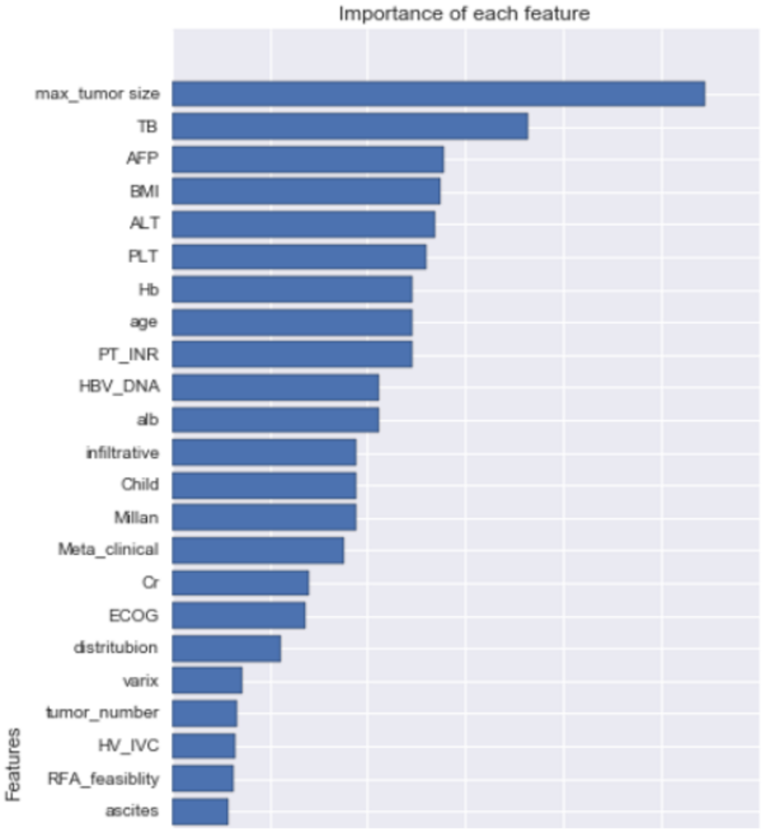

Supplementary Figure 2. Feature importance (Top 44) from random survival forest models. A) Resection. B) TACE. C) Sorafenib+Others.

A) Resection

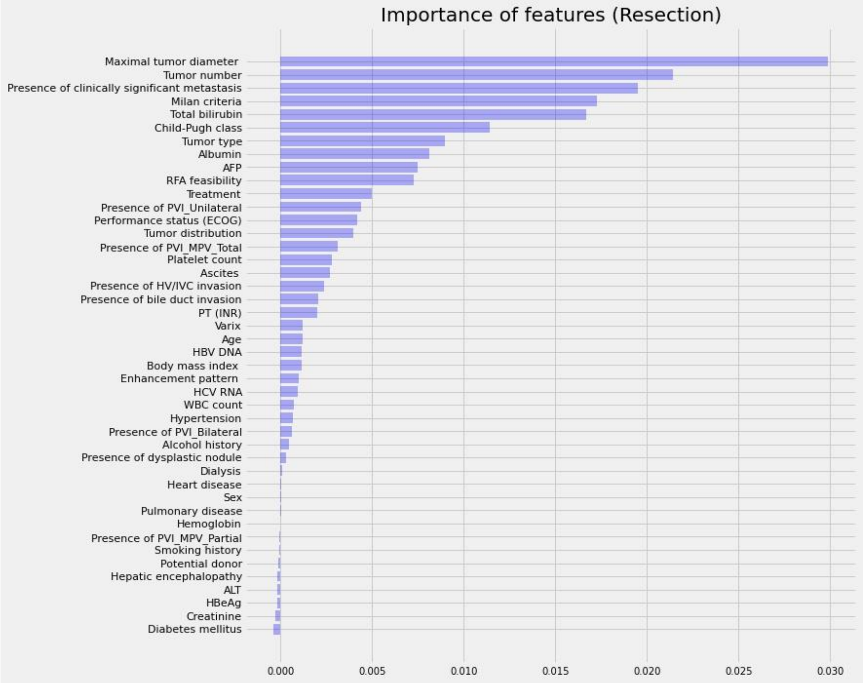

B) TACE

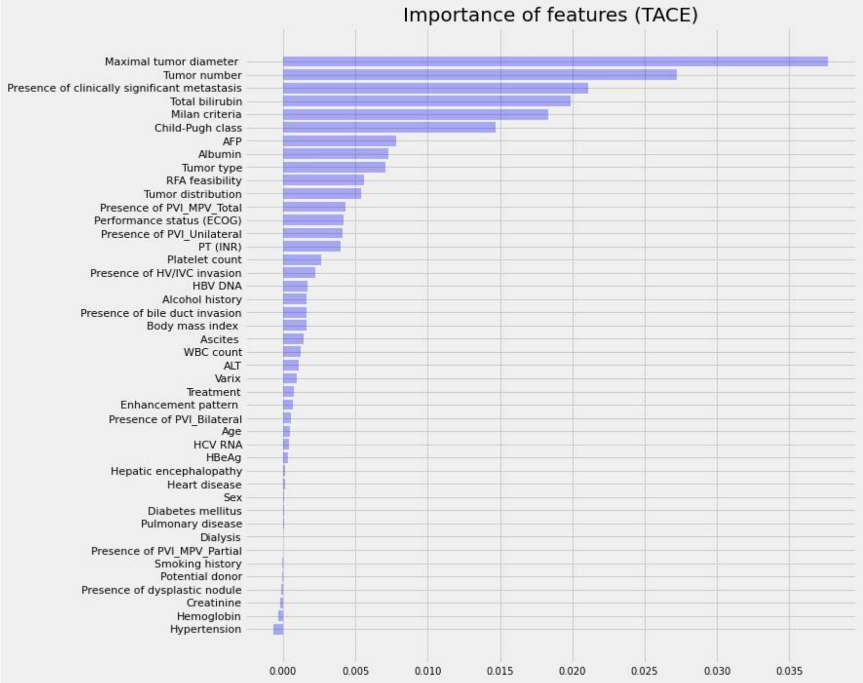

C) Sorafenib+Others

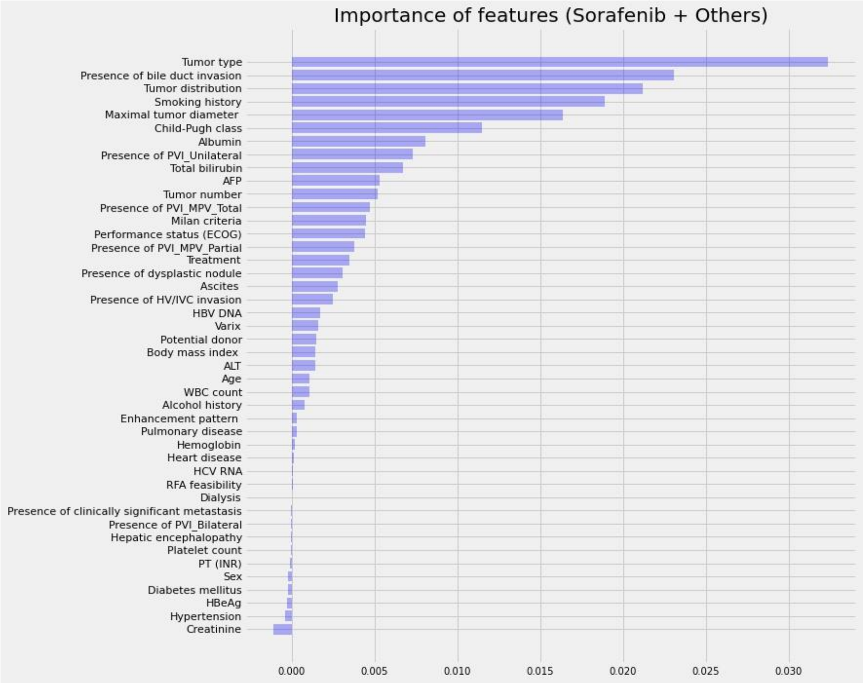

External validation datasets

Supplementary Table 2. Baseline characteristics of the patients in the external validation datasets.

		KUGH (n=138)	SNUBH (n=193)	SMC (n=439)	SNUH (n=224)	CMC (n=162)	SH (n=148)	CUH (n=171)	IUH (n=275)
Age, year		59.2 (36-84)	60.4 (32-89)	57 (29-81)	59.7 (29-85)	57.5 (29-85)	59.1 (27-89)	62.5 (29-88)	60.4 (29-93)
Gender	Male	116 (84.1)	153 (79.3)	365 (83.1)	174 (77.7)	128 (79.0)	114 (77.0)	132 (77.2)	230 (83.6)
	Female	22 (15.9)	40 (20.7)	74 (16.9)	50 (22.3)	34 (21.0)	34 (23.0)	39 (22.8)	45 (16.4)
ECOG performance status	0	32 (23.2)	103 (53.4)	407 (92.7)	165 (73.7)	80 (49.4)	75 (50.7)	19 (11.1)	103 (37.5)
	1 or 2	92 (66.7)	82 (42.5)	31 (7.1)	59 (26.3)	75 (46.3)	68 (45.9)	143 (83.6)	166 (60.4)
	3 or 4	14 (10.1)	8 (4.1)	1 (0.2)	0 (0)	7 (4.3)	5 (3.4)	9 (5.3)	6 (2.2)
Ascites	Absent	100 (72.5)	147 (76.2)	363 (82.7)	198 (88.4)	138 (85.2)	131 (88.5)	120 (70.2)	206 (74.9)
	Present	38 (27.5)	46 (23.8)	76 (17.3)	26 (11.6)	24 (14.8)	17 (11.5)	51 (29.8)	69 (25.1)
Varices	Absent	71 (51.4)	123 (63.7)	240 (54.7)	186 (83.0)	67 (41.4)	117 (79.1)	93 (54.4)	151 (54.9)
	Present	67 (48.6)	70 (36.3)	199 (45.3)	38 (17.0)	95 (58.6)	31 (20.9)	78 (45.6)	124 (45.1)
Child-Pugh class	A	96 (69.6)	151 (78.2)	381 (86.8)	184 (82.1)	129 (79.6)	120 (81.1)	112 (65.5)	180 (65.5)
	B	31 (22.5)	37 (19.2)	50 (11.4)	36 (16.1)	26 (16.0)	25 (16.9)	47 (27.5)	81 (29.5)
	C	11 (8.0)	5 (2.6)	8 (1.8)	4 (1.8)	7 (4.3)	3 (2.0)	12 (7.0)	14 (5.1)
Body mass index, kg/m^2		23.8 (17.5-35.5)	23.4 (15.5-31.6)	24.5 (16.4-39.1)	24.3 (17.4-34.9)	24.2 (17.3-36.6)	24.3 (16.8-35.6)	24.5 (17.5-41.2)	23.5 (13.8-34.2)
Tumour number	1	78 (56.5)	100 (51.8)	263 (59.9)	161 (71.9)	94 (58.0)	86 (58.1)	100 (58.5)	158 (57.5)
	2 or 3	21 (15.2)	33 (17.1)	109 (24.8)	43 (19.2)	35 (21.6)	40 (27.0)	38 (22.2)	49 (17.8)
	≥4	39 (28.3)	60 (31.1)	67 (15.3)	20 (8.9)	33 (20.4)	22 (14.9)	33 (19.3)	68 (24.7)
Maximal tumour size, cm		4.4 (0.7-10.0)	4.9 (0.9-10.0)	5.5 (1.0-10.0)	4.2 (0.7-10.0)	4.6 (0.7-10.0)	3.9 (0.9-10.0)	4.3 (1.0-10.0)	4.5 (1.0-10.0)
Distribution	Single segmental	70 (50.7)	95 (49.2)	201 (45.8)	108 (48.2)	97 (59.9)	69 (46.6)	105 (61.4)	125 (45.5)
	Unilobar	33 (23.9)	42 (21.8)	114 (26.0)	59 (26.3)	31 (19.1)	42 (28.4)	32 (18.7)	78 (28.4)
	Bilobar	35 (25.4)	56 (29.0)	124 (28.2)	57 (25.4)	34 (21.0)	37 (25.0)	34 (19.9)	72 (26.2)
Distant metastasis	Absent	128 (92.8)	175 (90.7)	379 (86.3)	207 (92.4)	154 (95.1)	136 (91.9)	158 (92.4)	257 (93.5)

	Present	10 (7.2)	18 (9.3)	60 (13.7)	17 (7.6)	8 (4.9)	12 (8.1)	13 (7.6)	18 (6.5)
Vascular invasion	Absent	101 (73.2)	147 (76.2)	269 (61.3)	171 (76.3)	137 (84.6)	129 (87.2)	152 (88.9)	217 (78.9)
	Unilateral	15 (10.9)	33 (17.1)	75 (17.1)	20 (8.9)	13 (8.0)	9 (6.1)	11 (6.4)	33 (12.0)
	Main or bilateral	22 (15.9)	13 (6.7)	95 (21.6)	33 (14.7)	12 (7.4)	10 (6.8)	8 (4.7)	25 (9.1)
RFA feasibility[†]	Feasible	44 (31.9)	36 (18.7)	76 (17.3)	100 (44.6)	52 (32.1)	20 (13.5)	35 (20.5)	116 (42.2)
	Non-feasible	94 (68.1)	157 (81.3)	363 (82.7)	124 (55.4)	110 (67.9)	128 (86.5)	136 (79.5)	159 (57.8)
Laboratory findings	AFP[*], ng/mL	27.3 (1.3-1339065.0)	15.7 (0.7-35000.0)	61.0 (1.3-200000.0)	34.0 (1.0-327700.0)	67.8 (1.0-639100.0)	23.5 (1.4-107212.6)	12.0 (0.7-60500.0)	34.9 (1.2-60500.0)
	Hemoglobin, g/dL	12.9 (4.8-18.1)	13.1 (5.0-17.3)	13.9 (6.6-19.4)	13.2 (7.5-16.9)	13.2 (5.9-18.0)	13.4 (7.4-17.8)	12.9 (6.3-17.5)	13.0 (3.3-22.0)
	Platelet count, x10^9/mm^3	140.3 (12.0-504.0)	167.8 (41.0-560.0)	150.5 (16.0-600.0)	136.4 (25.0-419.0)	148.1 (0.8-576.0)	143.0 (25.0-562.0)	143.5 (25.0-640.0)	150.2 (5.0-462.0)
	ALT, U/L	55.4 (8.0-412.0)	61.5 (7.0-716.0)	49.7 (8.0-431.0)	52.8 (0.1-446.0)	63.2 (9.0-1135.0)	36.9 (8.0-160.0)	45.5 (6.0-281.0)	50.1 (7.0-449.0)
	Total bilirubin, ml/dL	2.0 (0.3-25.7)	1.5 (0.2-22.5)	1.2 (0.2-21.7)	1.3 (0.3-28.7)	1.3 (0.3-24.8)	1.1 (0.2-5.2)	1.4 (0.2-17.3)	1.8 (0.3-38.2)
	Albumin, mg/dL	3.8 (2.2-4.8)	3.8 (1.7-5.0)	4.1 (2.4-5.2)	3.7 (1.6-4.9)	3.7 (1.2-4.9)	3.8 (2.0-5.0)	3.6 (1.4-4.7)	3.5 (0.8-5.0)
	Prothrombin time, INR	1.1 (0.8-2.4)	1.2 (0.9-3.5)	1.1 (0.9-2.2)	1.1 (0.8-1.7)	1.2 (0.9-12.9)	1.1 (0.9-1.7)	1.2 (0.9-2.8)	1.2 (0.9-3.4)
	Creatinine, mg/dL	0.8 (0.1-5.8)	0.8 (0.3-3.0)	0.9 (0.4-12.3)	0.9 (0.4-4.7)	1.0 (0.5-11.4)	0.9 (0.5-9.6)	0.9 (0.6-2.5)	1.1 (0-11.6)
Initial treatment	RFA or PEIT	25 (18.1)	21 (10.9)	64 (14.6)	45 (20.1)	35 (21.6)	10 (6.8)	16 (9.4)	24 (8.7)
	Resection	24 (17.4)	27 (14.0)	70 (15.9)	38 (17.0)	6 (3.7)	41 (27.7)	31 (18.1)	52 (18.9)
	TACE	60 (43.5)	107 (55.4)	157 (35.8)	129 (57.6)	105 (64.8)	78 (52.7)	110 (64.3)	143 (52.0)
	TACE combined with EBRT	0 (0)	6 (3.1)	107 (24.4)	0 (0)	1 (0.6)	0 (0)	0 (0)	0 (0)
	Sorafenib treatment	7 (5.1)	7 (3.6)	18 (4.1)	5 (2.2)	0 (0)	11 (7.4)	2 (1.2)	10 (3.6)
	Supportive care	22 (15.9)	25 (13.0)	23 (5.2)	7 (3.1)	15 (9.3)	8 (5.4)	12 (7.0)	46 (16.7)

Data are n (%), mean (SD), or [*]median (IQR) in parentheses.

KUGH Korea University Guro Hospital. *SNUBH* Seoul National University Bundang Hospital. *SMC* Samsung Medical Center. *SNUH* Seoul National University Hospital. *CMC* Catholic Medical Center. *SH* Severance Hospital. *CUH* Chung-ang University Hospital. *IUH* Inha University Hospital. *AFP* alpha-fetoprotein. *ALT* alanine aminotransferase. *EBRT* external beam radiotherapy. *ECOG* Eastern Cooperative Oncology Group. *INR* international normalized ratio. *PEIT* percutaneous ethanol injection. *RFA* radiofrequency ablation. *TACE* transarterial chemoembolization.

[†]RFA feasibility was defined as a size or location of the tumor to receive percutaneous RFA successfully without significant complications.

Supplementary Figure 3. Treatment distribution patterns by participating institutions.

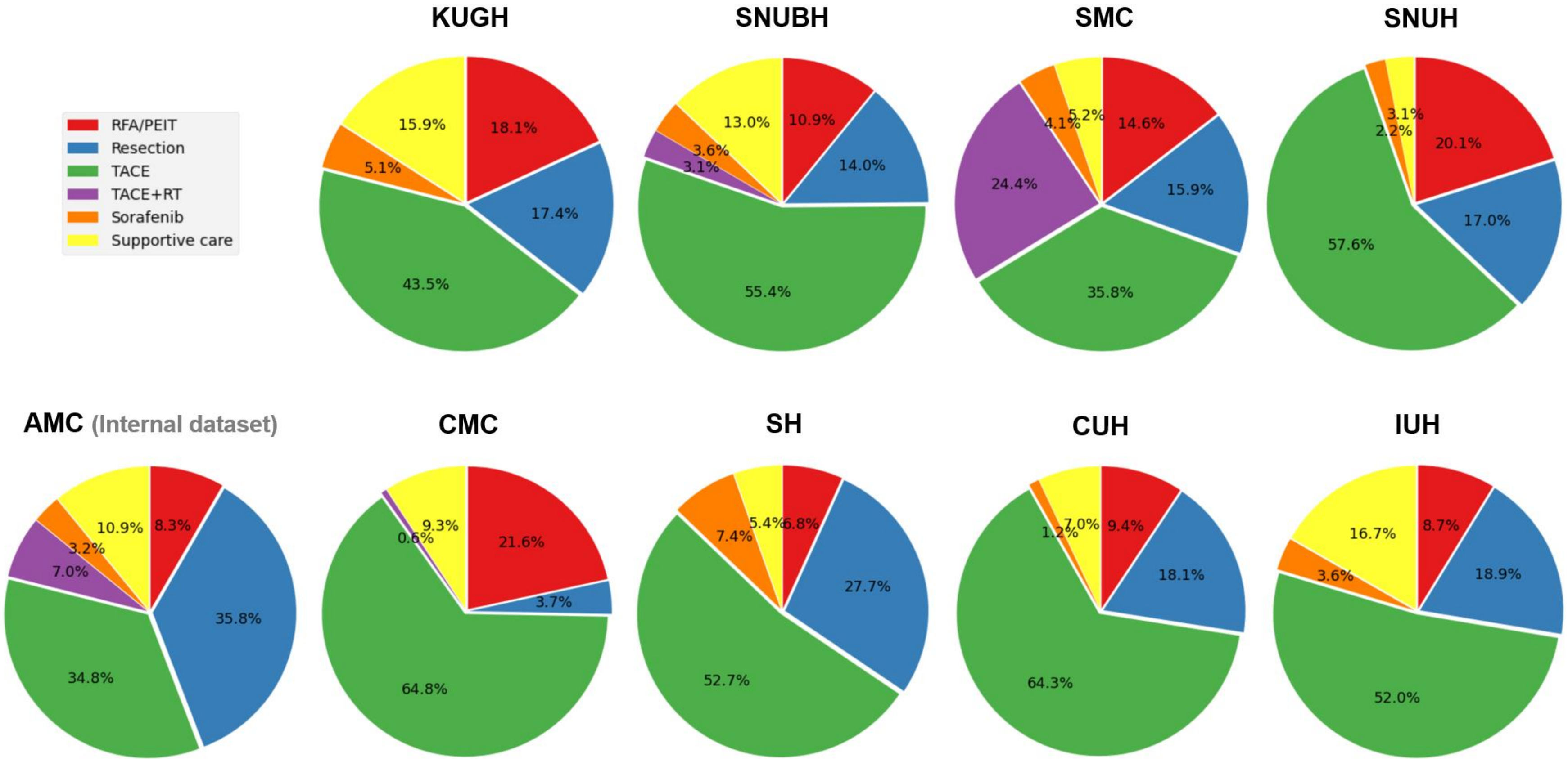

Model for treatment recommendation

We evaluated 19 different machine learning algorithms for the development of the treatment classification task: Logistic regression, Decision tree, Extra-trees, Random forest, Adaboost, Gradient boosting machine (gbm), Histogram-based gradient boosting, Xgboost, light gbm, CatBoost, Gaussian naive Bayes, Naive Bayes for multivariate Bernoulli models, Gaussian process classification, Linear discriminant analysis, Quadratic discriminant analysis, C-support vector machine, Multi-layer perceptron, K-nearest neighbors classifier, and K-means clustering. Scikit-Learn's version 0.23.2, Xgboost's version 1.5.0, catboost's version 1.0.1, and lightgbm's version 3.2.1 were used to construct these models.

All variables were normalized using Scikit-Learn's MinMaxScaler before training the machine learning algorithms. All classifiers underwent limited hyperparameter tuning during training. This implies that the iterative search for optimal hyperparameters to maximize performance with each variation of the training dataset was omitted. Most classifiers utilized the default hyperparameters as indicated below, with the only exception being the random state for result reproducibility. However, in some classifiers, empirical changes were made to the hyperparameters (e.g., using 'linear' kernel instead of the default 'radial basis function (rbf)' kernel in C-Support Vector Classifier), and these changes were fixed in a pre-determined state throughout the experiments.

ML algorithms	Hyperparameter
Logistic regression	LogisticRegression(random_state=999)<br>RidgeClassifier(random_state=999)<br>SGDClassifier(random_state=999)
Decision tree	DecisionTreeClassifier(random_state=999)
Extra-trees	ExtraTreesClassifier(random_state=999)
Random forest	RandomForestClassifier(random_state = 999)
Adaboost	AdaBoostClassifier(random_state=999)

Gradient boosting machine	GradientBoostingClassifier(random_state=999)
Histogram-based gradient boosting	HistGradientBoostingClassifier(random_state=999)
Xgboost	xgb.XGBClassifier(random_state=999, verbosity=0)
light gbm	LGBMClassifier(random_state=999, verbosity=-1, force_row_wise=True)
CatBoost	CatBoostClassifier(random_state=999, verbose=0)
Gaussian naive Bayes (NB)	GaussianNB()
NB for multivariate Bernoulli models	BernoulliNB()
Gaussian process classification	GaussianProcessClassifier(kernel=1.0*RBF(1.0), random_state=999)
Linear discriminant analysis	LinearDiscriminantAnalysis()
Quadratic discriminant analysis	QuadraticDiscriminantAnalysis()
C-support vector machine	svm.SVC(random_state=999, kernel='linear')<br>svm.SVC(random_state=999, probability=True)
Multi-layer perceptron	MLPClassifier(random_state=999)
K-nearest neighbors classifier	KNeighborsClassifier(n_neighbors=6)
K-means clustering	KMeans(n_clusters=6, random_state=999)

After evaluating all classifiers using stratified five-fold cross-validation, the classifiers were sorted based on mean accuracy. The top-performing three, five, and seven classifiers were then selected to train the ensemble voting machine. The ensemble voting classifier is an algorithm that combines the predictions of multiple individual classifiers to make the final prediction. These classifiers are independently trained using various learning algorithms based on the same dataset. It is primarily used for classification problems and can be categorized into hard voting and soft voting based on the way the final prediction is made by considering the predictions of each individual classifier. We have configured the voting mechanism to use the ‘soft voting’, whereby the class label prediction is derived based on the argmax of the sum of the predicted probabilities generated by each classifier.

The ensemble voting machines trained with the top-performing three, five, and seven classifiers were compared to the top-performing individual classifier itself. As the number of classifiers used in the ensemble voting machine increased, a slight improvement in

performance was observed. However, there were no significant differences in performance according to the number of classifiers (Supplementary Table 3). To avoid an excessive increase in complexity, we ultimately applied an ensemble voting machine comprising the top five performing classifiers.

Supplementary Table 3. Performance in terms of the number of classifiers composing voting classifier.

Dataset	No. of classifiers	Performance					
		Accuracy	Recall	Precision	F1	Kappa	MCC
Internal dataset	Top 1	65.45 (3.49)	51.09 (4.26)	64.79 (3.71)	64.32 (3.20)	51.41 (4.79)	51.75 (4.93)
	Top 3	67.06 (2.16)	49.64 (2.88)	64.89 (2.01)	65.34 (1.96)	53.01 (2.97)	53.39 (3.04)
	Top 5	67.27 (2.94)	52.22 (4.67)	65.82 (2.68)	65.93 (2.70)	53.85 (4.21)	54.22 (4.34)
	Top 7	67.27 (2.96)	53.04 (3.94)	65.93 (2.96)	66.05 (2.78)	54.12 (3.95)	54.46 (4.10)

Supplementary Table 4. Performance of ensemble voting classifier vs. cascaded random forest model.

Dataset	Model	Performance					
		Accuracy	Recall	Precision	F1	Kappa	MCC
Internal dataset	Cascaded	63.42 (3.74)	51.27 (2.51)	63.02 (3.52)	62.91 (3.54)	49.51 (4.92)	49.64 (4.96)
	Ensemble	67.27 (2.94)	52.22 (4.67)	65.82 (2.68)	65.93 (2.70)	53.85 (4.21)	54.22 (4.34)
External dataset	Cascaded	54.33 (4.29)	42.56 (4.79)	66.07 (5.06)	56.51 (3.04)	36.68 (5.97)	39.16 (5.37)
	Ensemble	55.34 (6.09)	41.68 (3.88)	64.14 (5.11)	56.82 (4.61)	36.30 (7.89)	38.33 (7.38)

Supplementary Table 5. Performance of individual training vs. external validation.

Center	No. of patients	Individual training		External validation	
		Accuracy	Recall	Accuracy	Recall
KUGH	138	66.03 (5.81)	52.87 (3.49)	61.59	44.77
SNUBH	193	70.49 (3.68)	42.03 (3.63)	56.99	38.78
SMC	439	65.83 (4.30)	49.72 (4.97)	52.62	44.09
SNUH	224	62.10 (8.85)	39.64 (9.98)	52.68	44.69
CMC	162	71.57 (5.74)	45.33 (11.16)	43.21	39.62
SH	148	58.09 (6.31)	43.99 (11.70)	60.14	38.05
CUH	171	74.86 (6.58)	53.87 (12.96)	63.16	47.65
IUH	275	63.27 (5.91)	45.06 (7.13)	52.36	35.8
Average	219	66.53 (5.90)	46.56 (8.13)	55.34 (6.09)	41.68 (3.88)

Data are mean (SD) in parentheses.
KUGH Korea University Guro Hospital. *SNUBH* Seoul National University Bundang Hospital. *SMC* Samsung Medical Center. *SNUH* Seoul National University Hospital. *CMC* Catholic Medical Center. *SH* Severance Hospital. *CUH* Chung-ang University Hospital. *IUH* Inha University Hospital.

Model for survival prediction

We trained the random survival forest (RSF) algorithm from the sksurv's 0.15.0 version to predict individual post-treatment survival. The random survival forest algorithm is an extension of the traditional random forest algorithm to handle survival data. The RSF model is used to model the survival function, which estimates the probability of an event occurring at or after a given time. Similar to the ensemble voting machine, we utilized the default hyperparameters as indicated below, with the only exception being the random state for the reproducibility of the results.

Hyperparameter	Default value
criterion	logrank
n_estimators	100
max_depth	None
min_samples_split	6
min_samples_leaf	3
min_weight_fraction_leaf	0
max_features	sqrt
max_leaf_nodes	None
bootstrap	True
oob_score	False
n_jobs	None
random_state	999
verbose	0
warm_start	False
max_samples	None

Calibration of model

Model calibration is the process of adjusting the predicted probabilities of a classifier to achieve more consistent and reliable probability estimation. By performing calibration across various subsets or regions of the data, model calibration reduces systemic biases and enhances the robustness of predictions. We calibrated the ensemble voting classifier using the CalibratedClassifierCV class from the sklearn.calibration package version 0.23.2. CalibratedClassifierCV combines cross-validation and calibration to adjust the predicted probabilities of the model. The method to use for calibration was 'sigmoid', which corresponds to a logistic regression model. The calibration process was conducted using 3-fold cross-validation.

Supplementary Table 6. Performance of non-calibrated model vs. calibrated model

Dataset	Calibration	Options	Performance					
			Accuracy	Recall	Prec.	F1	Kappa	MCC
Internal dataset	Pre-calibration	1st	67.27 (2.94)	52.22 (4.67)	65.82 (2.68)	65.93 (2.70)	53.85 (4.21)	54.22 (4.34)
		2nd	87.27 (2.25)	71.24 (2.90)	84.84 (2.19)	85.74 (2.17)	82.17 (3.17)	82.39 (3.20)
	Post-calibration	1st	65.99 (2.68)	50.35 (3.45)	64.45 (2.65)	64.60 (2.46)	51.97 (3.53)	52.32 (3.66)
		2nd	86.52 (2.84)	69.52 (4.24)	84.66 (3.31)	85.00 (2.92)	81.00 (4.11)	81.31 (4.06)
External dataset	Pre-calibration	1st	55.34 (6.09)	41.68 (3.88)	64.14 (5.11)	56.82 (4.61)	36.30 (7.89)	38.33 (7.38)
		2nd	86.06 (3.10)	64.49 (8.16)	88.38 (3.77)	85.83 (3.24)	78.13 (4.50)	78.69 (4.36)
	Post-calibration	1st	56.65 (4.98)	45.28 (7.39)	66.17 (5.71)	58.60 (4.09)	38.33 (6.24)	40.50 (5.69)
		2nd	85.29 (1.65)	62.24 (7.32)	85.92 (3.59)	84.92 (2.44)	76.74 (2.89)	77.33 (2.82)

Supplementary Figure 4. Calibration plot. A) Pre-calibration. B) Post-calibration.

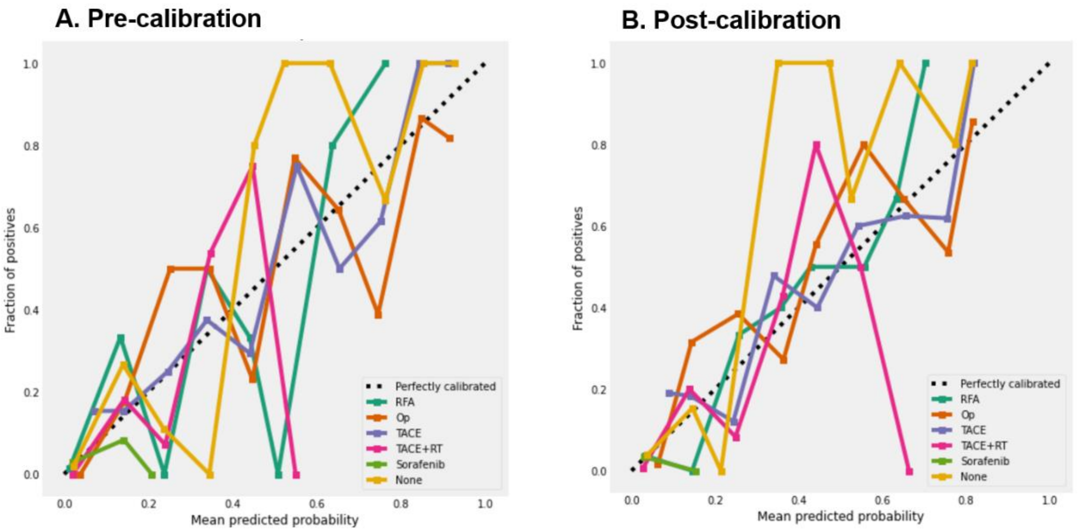

Propensity score matching

The objective of propensity score matching (PSM) is to create a control group that closely resembles the treatment group in terms of observed characteristics, enabling a more accurate estimation of treatment effects. In order to assess how the performance of the model changes among patients with similar conditions, we attempted additional experiments using propensity score matching (PSM). We aimed to extract Resection and TACE groups with similar characteristics through PSM and evaluate the results of our CDSS model. Due to 80% of the internal dataset already being used for model training and only a limited number of patients remaining in the test set (n=67 for the Resection group and n=65 for TACE group), we conducted PSM for the TACE and surgery groups (n=289 for the Resection group and n=889 for TACE group) in the external validation datasets (Supplementary Table 7).

We estimated the propensity scores using a logistic regression model. The treatment (Resection vs. TACE) was used as the outcome variable, while the 20 observed covariates were used as predictors. The estimated propensity scores were calculated for each individual in the dataset using the predicted probability from the logistic regression model. We utilized propensity scores to match 1:1 the closest resection patients with TACE patients without duplication. Subsequently, we validated the treatment prediction model using these matched groups. Experimental results revealed that in the matched resection group, there was an increased proportion of TACE predictions as the initial treatment compared to the unmatched resection group, while in the matched TACE group, a higher proportion of resection predictions as the initial treatment was observed compared to the unmatched TACE group. This effect was particularly prominent in the matched TACE group (Supplementary Figure 5).

In order to understand the underlying cause, we conducted feature analysis using the t-distributed stochastic neighbor embedding (t-SNE) as a feature reduction technique. We performed feature reduction for the TACE group in the external dataset used for testing, separately for cases where TACE was correctly predicted and cases where it was misclassified

as Resection. Additionally, we conducted the same analysis for the TACE and Resection groups in the internal dataset used for model training (Supplementary Figure 6A). Furthermore, the same analysis was performed for the matched TACE group as well (Supplementary Figure 6B). Through t-SNE visualization, we observed that the features of the TACE group in the external dataset, which were misclassified as Resection, were closely clustered with the features from the Resection group in the internal dataset used for model training. This observation was more pronounced in the matched TACE group. Our feature analysis suggests that patients with similar features, but belonging to different institutions, may receive different treatments.

Supplementary Table 7. Baseline characteristics of the patients in the external validation datasets.

		Resection (n=289)	TACE (n=889)	*p value*	Matched resection (n=225)	Matched TACE (n=225)	*p value*
Age, year		56.8 (27~89)	59.8 (29~89)	<0.0001	58.5 (32~89)	58.0 (29~88)	0.6361
ECOG performance status	0	206 (71.3)	444 (49.9)	NaN	153 (68.0)	155 (68.9)	NaN
	1 or 2	83 (28.7)	433 (48.7)		72 (32.0)	69 (30.7)	
	3 or 4	0 (0)	12 (1.3)		0 (0)	1 (0.4)	
Ascites	Absent	277 (95.8)	730 (82.1)	<0.0001	213 (94.7)	204 (90.7)	0.1480
	Present	12 (4.2)	159 (17.9)		12 (5.3)	21 (9.3)	
Varices	Absent	248 (85.8)	527 (59.3)	<0.0001	186 (82.7)	180 (80.0)	0.5452
	Present	41 (14.2)	362 (40.7)		39 (17.3)	45 (20.0)	
Child-Pugh class	A	276 (95.5)	689 (77.5)	<0.0001	213 (94.7)	207 (92.0)	0.5104
	B	12 (4.2)	183 (20.6)		11 (4.9)	16 (7.1)	
	C	1 (0.3)	17 (1.9)		1 (0.4)	2 (0.9)	
Body mass index, kg/m^2		24.0 (16.1~35.5)	24.2 (15.5~41.2)	0.2212	24.0 (16.1~35.5)	23.8 (17.4~32.2)	0.3214
Tumour number	1	243 (84.1)	444 (49.9)	<0.0001	182 (80.9)	160 (71.1)	0.0514
	2 or 3	40 (13.8)	247 (27.8)		37 (16.4)	55 (24.4)	
	≥4	6 (2.1)	198 (22.3)		6 (2.7)	10 (4.4)	
Maximal tumour size, cm		3.9 (1.0~10.0)	4.6 (0.7~10.0)	0.0383	4.0 (1.0~10.0)	4.1 (0.7~10.0)	0.2922
Distribution	Single segmental	204 (70.6)	384 (43.2)	<0.0001	150 (66.7)	141 (62.7)	0.4052
	Unilobar	62 (21.5)	253 (28.5)		53 (23.6)	53 (23.6)	
	Bilobar	23 (8.0)	252 (28.3)		22 (9.8)	31 (13.8)	
Distant metastasis	Absent	285 (98.6)	827 (93.0)	0.0006	221 (98.2)	219 (97.3)	0.7491
	Present	4 (1.4)	62 (7.0)		4 (1.8)	6 (2.7)	
Vascular invasion	Absent	278 (96.2)	729 (82.0)	NaN	214 (95.1)	212 (94.2)	NaN
	Unilateral	11 (3.8)	94 (10.6)		11 (4.9)	10 (4.4)	
	Main or bilateral	0 (0)	66 (7.4)		0 (0)	3 (1.3)	
RFA feasibility†	Feasible	78 (27.0)	158 (17.8)	0.0009	55 (24.4)	43 (19.1)	0.2090

	Non-feasible	211 (73.0)	731 (82.2)		170 (75.6)	182 (80.9)	
Laboratory findings	AFP*, ng/mL	14.9 (0.8~200000)	31.1 (0.7~327700)	0.0001	18.4 (1.1~200000)	15.4 (0.7~268862)	0.2710
	Hemoglobin, g/dL	14.3 (6.8~18.0)	13.1 (4.8~18.7)	<0.0001	14.1 (6.8~17.7)	14.1 (8.6~18.1)	0.4413
	Platelet count, $x10^9/mm^3$	169.2 (40~463)	139.8 (9~600)	<0.0001	159.3 (40~463)	164.1 (35~600)	0.4166
	ALT, U/L	43.3 (8~360)	50.3 (2~716)	0.0016	45.3 (8~360)	42.2 (2~308)	0.4264
	Total bilirubin, ml/dL	0.9 (0.2~5.2)	1.2 (0.2~14.9)	<0.0001	0.9 (0.2~5.2)	1.0 (0.3~12.1)	0.1908
	Albumin, mg/dL	4.2 (2.4~5.2)	3.7 (0.8~5.0)	<0.0001	4.1 (2.4~5.2)	4.1 (2.4~4.9)	0.2059
	Prothrombin time, INR	1.0 (0.8~1.9)	1.1 (0.8~3.4)	<0.0001	1.1 (0.8~1.9)	1.1 (0.8~2.4)	0.0190
	Creatinine, mg/dL	0.9 (0.0~2.5)	0.9 (0.0~11.4)	0.1189	0.9 (0.0~2.5)	0.8 (0.3~1.6)	0.0160

Data are n (%), mean or *median (range) in parentheses. p values were calculated using the χ^2 test or Student t-test or Mann–Whitney U test to compare the Resection group with the TACE group and the Matched resection group with the Matched TACE group.

AFP alpha-fetoprotein. *ALT* alanine aminotransferase. *EBRT* external beam radiotherapy. *ECOG* Eastern Cooperative Oncology Group. *INR* international normalized ratio.

†RFA feasibility was defined as a size or location of the tumor to receive percutaneous RFA successfully without significant complications.

Supplementary Figure 5. Normalized confusion matrix for the treatment classification model. A) Patients who underwent resection, B) Patients who underwent resection and were matched to the TACE group, C) Patients who underwent TACE, D) Patients who underwent TACE and were matched to the resection group.

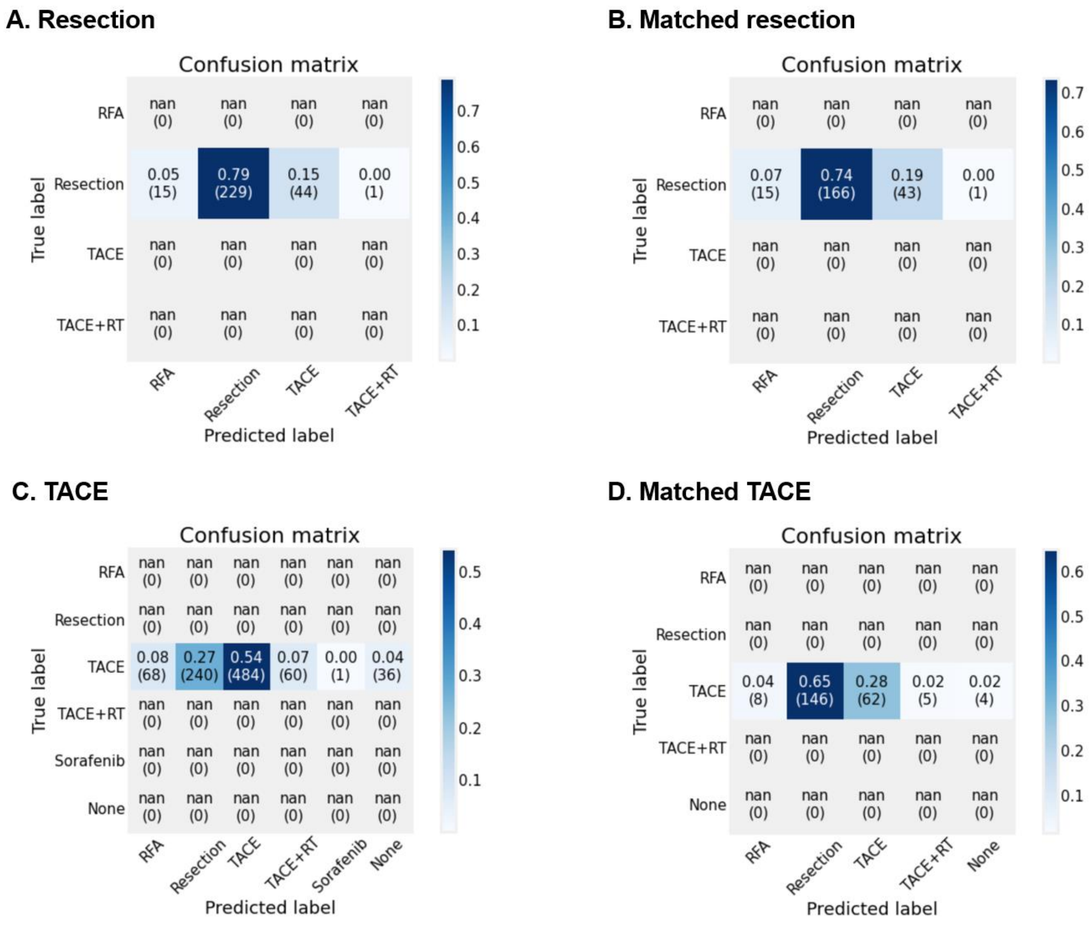

Supplementary Figure 6. Feature analysis using feature reduction with t-distributed stochastic neighbor embedding (t-SNE) for cases with correct (Treatment prediction = TACE) and incorrect predictions (Treatment prediction = Resection). A) TACE group. B) Matched TACE group.

A) TACE group

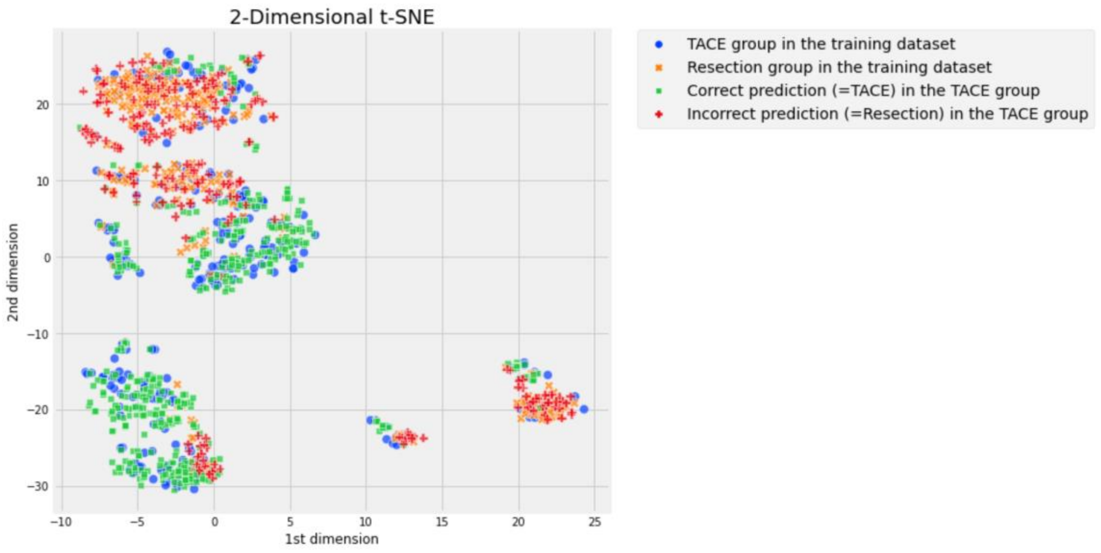

B) Matched TACE group

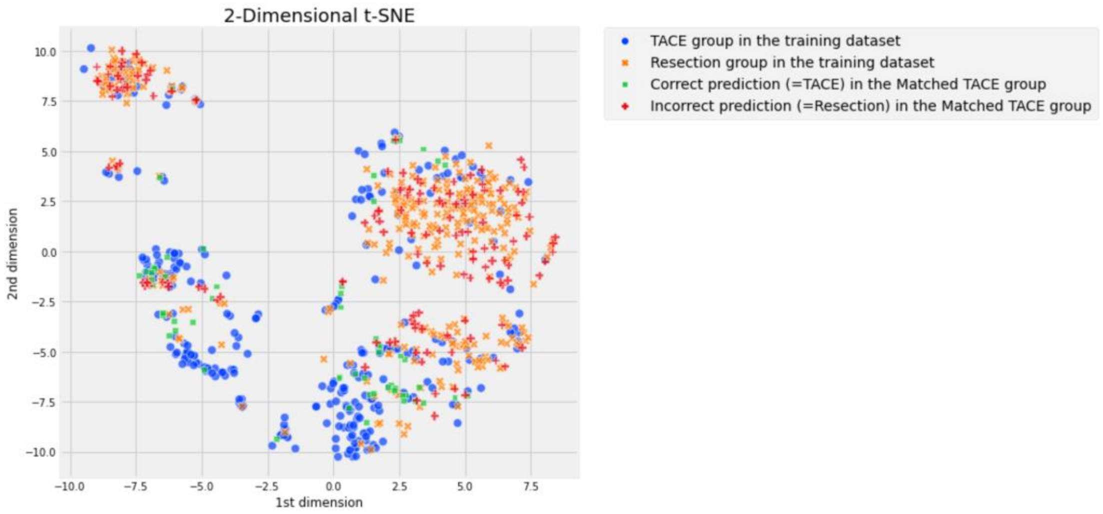

Model deployment

Supplementary Figure 7. Issues on model training and deployment

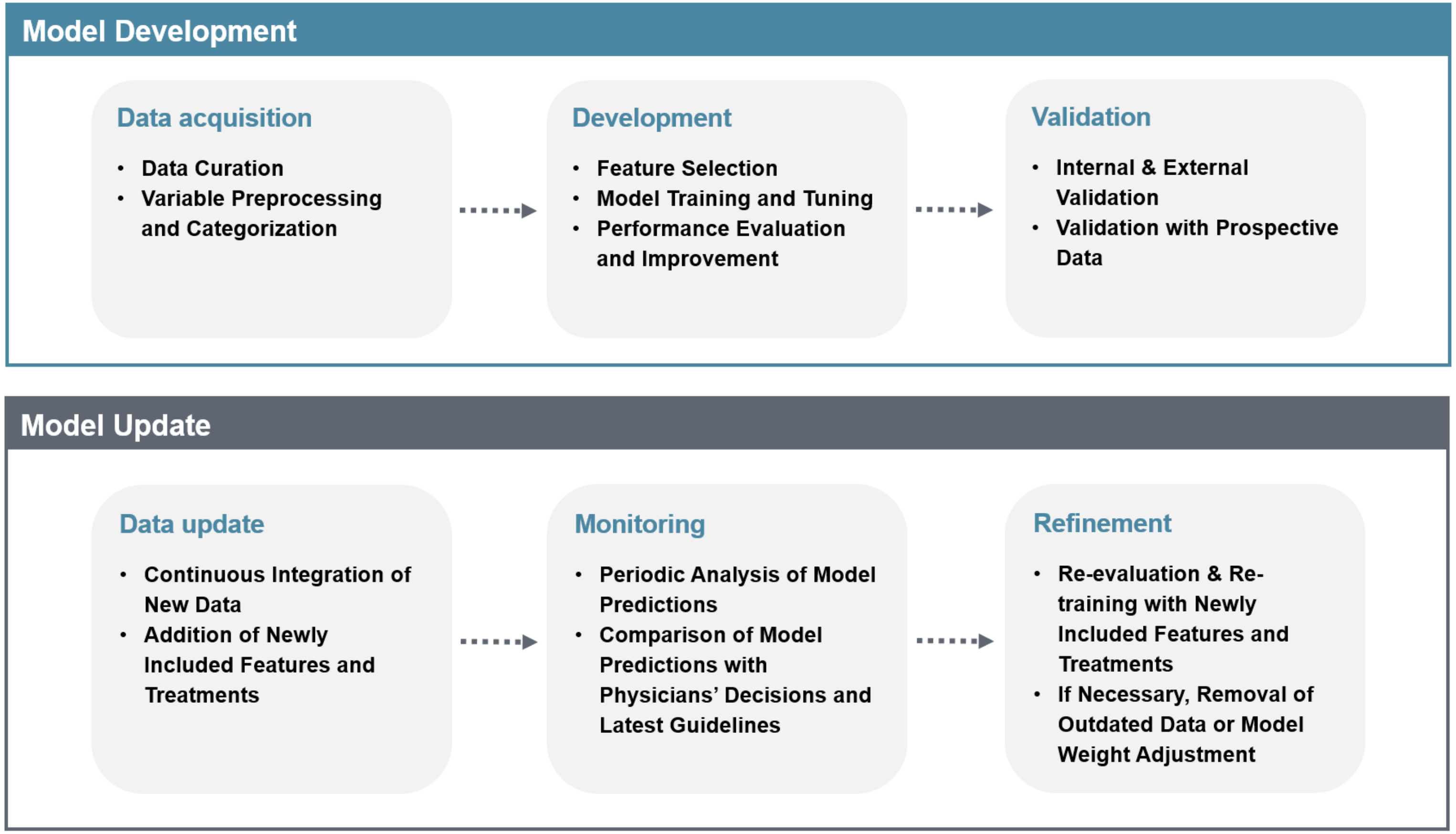

Supplement: Supplementary file 1 — Supplementary Information [file 41746_2023_976_MOESM1_ESM.pdf]
